# Mini review of photoacoustic clinical imaging: a noninvasive tool for disease diagnosis and treatment evaluation

**DOI:** 10.1117/1.JBO.29.S1.S11522

**Published:** 2024-01-16

**Authors:** Huazhen Liu, Ming Wang, Fei Ji, Yuxin Jiang, Meng Yang

**Affiliations:** Chinese Academy of Medical Sciences and Peking Union Medical College, Peking Union Medical College Hospital, Department of Ultrasound, Beijing, China

**Keywords:** photoacoustic imaging, tumor, autoimmune disease, inflammatory disease, endocrine disorder

## Abstract

**Significance:**

Photoacoustic (PA) imaging is an imaging modality that integrates anatomical, functional, metabolic, and histologic insights. It has been a hot topic of medical research and draws extensive attention.

**Aim:**

This review aims to explore the applications of PA clinical imaging in human diseases, highlighting recent advancements.

**Approach:**

A systemic survey of the literature concerning the clinical utility of PA imaging was conducted, with a particular focus on its application in tumors, autoimmune diseases, inflammatory conditions, and endocrine disorders.

**Results:**

PA imaging is emerging as a valuable tool for human disease investigation. Information provided by PA imaging can be used for diagnosis, grading, and prognosis in multiple types of tumors including breast tumors, ovarian neoplasms, thyroid nodules, and cutaneous malignancies. PA imaging facilitates the monitoring of disease activity in autoimmune and inflammatory diseases such as rheumatoid arthritis, systemic sclerosis, arteritis, and inflammatory bowel disease by capturing dynamic functional alterations. Furthermore, its unique capability of visualizing vascular structure and oxygenation levels aids in assessing diabetes mellitus comorbidities and thyroid function.

**Conclusions:**

Despite extant challenges, PA imaging offers a promising noninvasive tool for precision disease diagnosis, long-term evaluation, and prognosis anticipation, making it a potentially significant imaging modality for clinical practice.

## 1 Introduction

Photoacoustic (PA) imaging is an emerging medical imaging technique that enables detailed visualization of tissue structures and functional information.^1^^,^^2^ The basis of PA imaging lies in the PA effect, a physical phenomenon in which acoustic waves are generated through light absorption.^1^^,^^3^^,^^4^ The transformation of PA effects from a theoretical concept to a valuable imaging modality has taken over a century of development.^5^^,^^6^ In recent years, numerous research groups and companies have developed medical devices and processing systems available for human imaging.^1^^,^^2^^,^^6^^–^^8^ Typically, PA imaging devices consist of several essential components, including a short-pulsed laser for generating PA signals, an ultrasonic transducer or transducer array for detecting PA signals, a data-acquisition system for amplifying and digitizing PA signals, and a computer for forming image.^1^^,^^6^

**Fig.1:**
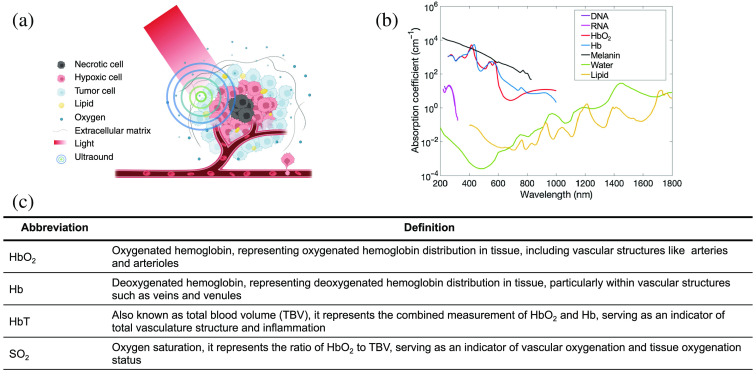
Schematic diagram of oxygen distribution in solid tumor and PA imaging principles. (a) The peripheral distribution of vasculature in the tumor, revealing decreased oxygen levels in the tumor’s center/core, resulting in necrosis. PA imaging involves the absorption of pulsed light by tissue, leading to a rise in temperature and subsequent US wave generation, which is subsequently detected. (b) Absorption spectra of endogenous contrasts. (c) Common terms and definitions used in the detection of hemoglobin in PA imaging. Panel a is created with Ref. 9. Panel b is reprinted with permission from Ref. 10, under the terms of Creative Commons CC BY license.

As PA imaging merges laser-induced optical excitation with ultrasound (US) detection, it has advantages for clinical applications as follows: (1) PA imaging allows for noninvasive functional, metabolic, and histological imaging using endogenous contrasts such as hemoglobin [oxygenated hemoglobin (${\mathrm{HbO}}_{2}$), deoxygenated hemoglobin (Hb), total hemoglobin (HbT), also known as total blood volume (TBV)], lipids, and water (Fig. 1);^10^^–^^16^ (2) PA imaging surpasses the penetration limitations of conventional optical methods, making it superior for deep tissue imaging compared with pure optical imaging techniques;^17^ (3) PA imaging can integrate with other imaging modalities, facilitating multimodal approaches that enhance diagnostic accuracy;^18^ and (4) PA imaging offers portability in a safe way, making it suitable for longitudinal patient monitoring.^1^^,^^19^^–^^22^ Owing to these advantages, PA imaging has received significant attention in medical research.^7^^,^^13^^,^^23^

Here, we review promising clinical applications of PA imaging, with a specific focus on tumors, autoimmune diseases, inflammatory conditions, and endocrine disorders. The literature reporting human imaging studies, especially those involving stand-alone multispectral optoacoustic tomography (MSOT) and hybrid PA and US systems, was primarily reviewed. The aim of this review is to highlight the current advancements in PA imaging. The concluding segment will briefly discuss the challenges that still need to be addressed in PA imaging.

## 2 Tumor

Malignant tumor is the primary cause of mortality and acts as one of the most important barriers to increasing life expectancy.^24^^,^^25^ Updated epidemiological data indicate that there were ∼19.3  million new cases of cancer and nearly 10.0 million cancer-related deaths worldwide.^25^ Hypoxia is a significant hallmark in solid tumors, resulting from the imbalance between the tumor cell proliferation and the available blood supply (Fig. 1).^26^^,^^27^ This intricate hypoxia microenvironment further leads to the regulation of molecular signaling cascades responsible for fundamental cellular processes, including cell cycle progression and angiogenesis. Tumor-associated hypoxia is related to oncological pathophysiology, including disease progression, therapeutic resistance, and poor outcome.^28^^–^^30^

PA imaging emerges as a compelling paradigm for visualizing the intricate landscape of tumor hypoxia, thereby facilitating early diagnosis and precise evaluation of the tumor.^27^^,^^31^^,^^32^ Its efficacy has been explored in diverse tumors, including breast tumors, ovarian neoplasms, thyroid nodules, and cutaneous malignancies.

### 2.1 Breast Tumor

Breast tumor is the most common cancer and stands as the primary contributor to cancer-related mortality among women.^25^ The overlap in the morphological features between benign and malignant breast tumors poses a great clinical challenge.

PA imaging is a valuable modality for elucidating functional intricacies within breast tumors.^33^ In 2011, PA tomography was applied for imaging the vascularity and oxygenation of invasive breast tumors and ductal carcinoma *in situ*.^34^ The lesions exhibited an average oxygenation saturation (${\mathrm{SO}}_{2}$) of 78.6%, along with a concentration of HbT measuring 207  μM.^34^ Handheld US combined with MSOT also shows the ability for breast tumor imaging.^35^^–^^41^ MSOT unveiled a pattern of breast cancer characterized by rim enhancement and a diminished core PA signal, evident in both $\mathrm{Hb}/{\mathrm{HbO}}_{2}$ and TBV images. This pattern correlated with peripheral perfusion but reduced core blood perfusion within the tumor. The findings were validated through postoperative anti-CD31 staining.^35^^,^^36^

The precision of breast tumor diagnosis may be improved as PA imaging could provide functional information. The analysis using MSOT revealed significant distinctions in TBV between tumors and normal tissues. Interestingly, disruptions were observed in the water and fat lipid layers in cancerous tissues, potentially suggesting the infiltration of tumors into the adipose tissue.^35^ An integrated MSOT/US imaging system exhibited significantly higher hemoglobin signals in invasive carcinoma compared with healthy tissues, indicating increased perfusion in tumor and the tumor environment.^37^

**Fig. 2:**
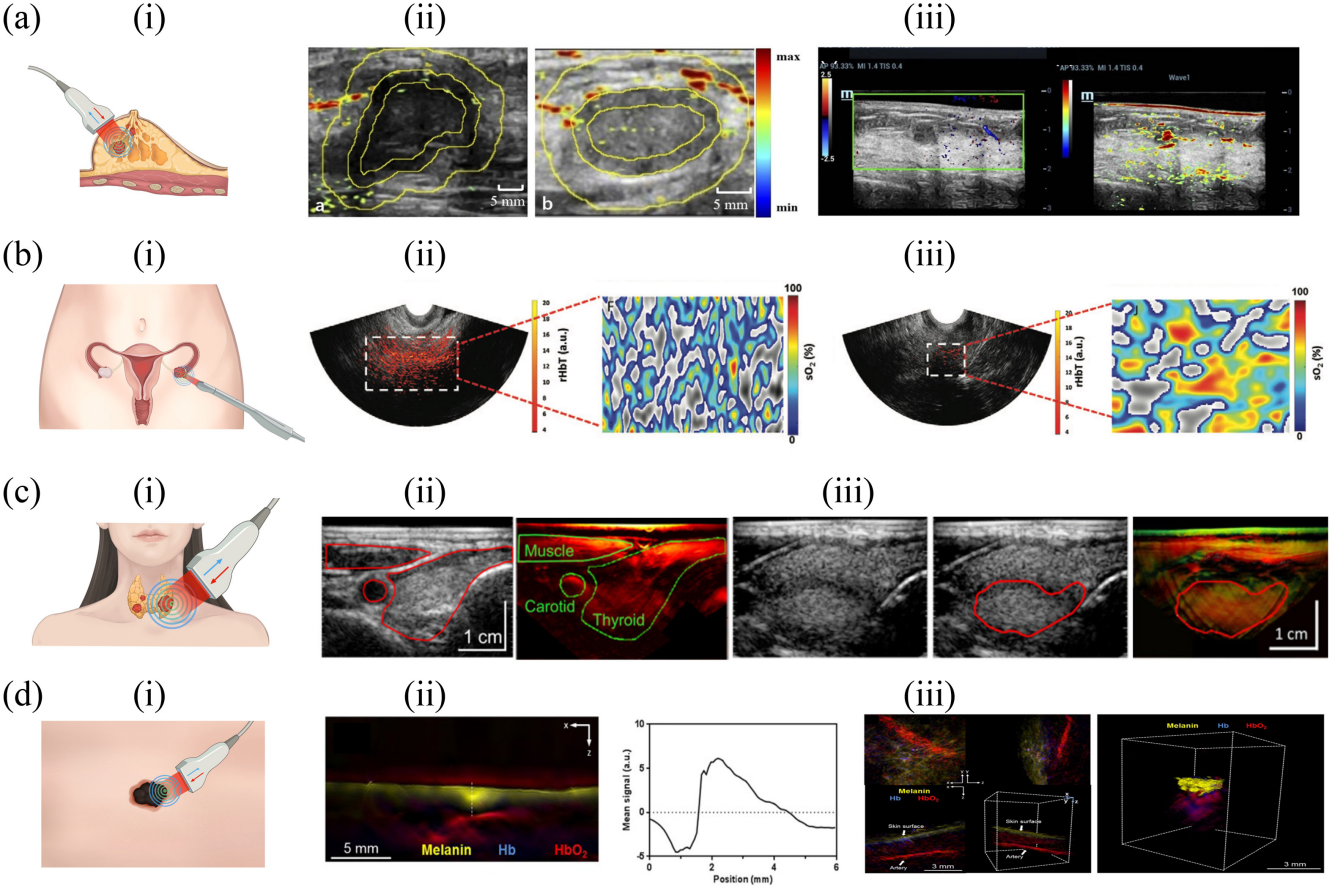
Representative *in vivo* PA imaging for tumors. (a) Breast tumor PA imaging: (i) a hand-held optoacoustic unit for breast tumor PA imaging. (ii) Left: PA/US modality imaging of a malignant breast lesion (F, 40 years), with limited PA signals in the central region and abundant signals in the peripheral and surrounding regions. Right: PA/US imaging of a benign breast lesion (F, 36 years) with abundant PA signals in all depicted regions. (iii) Intraductal papilloma (F, 65 years). Left: grayscale US with no blood signal indicated by color doppler flow imaging in the lesion. Right: abundant vessel signals indicated by PA. (b) Ovarian tumor PA imaging: (i) A hand-held optoacoustic unit for ovarian tumor PA imaging. Images of (ii) a 4.5 cm endometrioid adenocarcinoma (F, 63 years) and (iii) a 2.2 cm benign tumor (F, 63y). (ii) A coregistered US/PA image with extensive diffused vascular distribution (rHbT map) and an average ${\mathrm{SO}}_{2}$ in the region of interest (ROI) of 43.2% (${\mathrm{SO}}_{2}$ map). (iii) The scattered vascular distribution (rHbT map) and an average ${\mathrm{SO}}_{2}$ in the ROI of 58.2% (${\mathrm{SO}}_{2}$ map). (c) Thyroid nodule PA imaging: (i) A hand-held optoacoustic unit for thyroid nodule PA imaging. (ii) Thyroid MSOT imaging. Transversal US image (left) and MSOT image (right, showing HbT) of the thyroid gland and surrounding tissue. (iii) MSOT imaging of the benign thyroid nodule. (d) Skin cancer PA imaging: (i) A hand-held optoacoustic unit for skin cancer PA imaging. (ii) MSOT imaging of the superficial basal cell carcinoma acquired by the 2D probe and its distribution along the depth dimension. (iii) Left: 3D MSOT imaging of a viral wart, which identified the large artery by the characteristics of ${\mathrm{HbO}}_{2}$ absorption. Right: 3D MSOT imaging of a representative basal cell carcinoma lesion. Melanin signals (yellow) were clustered at the top with strong hemoglobin signals underneath the carcinoma (Hb: blue, ${\mathrm{HbO}}_{2}$: red). Panel a-ii) is reprinted with permission from Ref. 40 © The Optical Society. Panel a-iii is reprinted with permission from Ref. 41 © The Optical Society. Panels b-ii and b-iii are reprinted with permission from Ref. 42 © RSNA. Panels c-ii and c-iii are reprinted with permission from Ref. 43, originally published in JNM, © SNMMI. Panels d-ii and d-iii are reprinted with permission from Ref. 44, under the terms of Creative Commons CC BY license.

Researchers have also explored various quantitative strategies for characterizing breast tumors. Using a three-dimensional (3D) PA/US functional imaging platform, researchers identified that invasive breast cancer (stage T1) exhibited a 7.7% lower average volumetric ${\mathrm{SO}}_{2}$ compared with benign tumors and a 3.9% decrease compared with healthy breast tissue, both statistics significantly. Employing an ${\mathrm{SO}}_{2}$ threshold of 78.2%, the model achieved high sensitivity (100%) but moderate specificity (62.5%), resulting in an area under the curve (AUC) of 0.81 for T1 invasive breast cancer differentiation.^39^ Further quantitative and semi-quantitative analysis focused on PA signal spatial distribution [Fig. 2(a)].^40^ The malignant group displayed significantly greater relative PA signal differences between surrounding/peripheral region and central regions compared with the benign group. A diagnostic model exhibited a strong diagnostic capacity for distinguishing malignancies from benign lesions (sensitivity: 80.8%, specificity: 85.7%, and AUC: 86.5%).^40^ Regarding breast intraductal lesions, intraductal lesions exhibited lower ${\mathrm{SO}}_{2}$ compared with benign lesions. A diagnostic model, which combined PA-indicated interval vessel data and ${\mathrm{SO}}_{2}$ scores, achieved an accuracy rate of 90% sensitivity and 87.5% specificity when discriminating intraductal lesions from benign lesions [Fig. 2(a)].^41^ Interestingly, the spatial distribution and spectral analysis of the PA signal may also help distinguish subtypes of breast cancer.^45^^,^^46^ Luminal breast cancers (both luminal A and luminal B) have exhibited higher external PA signals and lower internal PA signals than those of human epidermal growth factor receptor 2-positive and triple-negative cancers.^45^

The American College of Radiology Breast Imaging Report and Data System (BI-RADS) system is a recognized protocol for diagnosing breast conditions, with its primary emphasis on morphological features of lesions. The incorporation of PA/US enhances the specificity of breast tumor evaluation when compared with grayscale US in isolation.^47^ Subsequent investigations endeavored to incorporate the functional data from PA imaging to upgrade and downgrade BI-RADS scores for potentially malignant and benign lesions correspondingly. Benign tumors categorized as BI-RADS 4a could potentially be reclassified to lower categories, thereby decreasing the need for unnecessary biopsies and subsequent follow-up assessments. However, it is important to note that, in a small number of cases, there is a risk of inappropriate reclassification.^48^ In the case of intraductal lesions, the PA/US approach led to the upgrading of 40% breast intraductal lesions and the downgrading of 50% benign lesions compared with the BI-RADS system.^41^

### 2.2 Ovarian Tumor

Ovarian tumors encompass a spectrum of classifications, including benign, borderline, and malignant. Most ovarian malignancies are diagnosed with metastasis, contributing to high gynecological cancer mortality.^49^^,^^50^ Early differentiation of malignancies from benign/normal masses is important, offering opportunities for prompt multidisciplinary intervention.

Researchers at Washington University School of Medicine employed a transvaginal PA/US system to differentiate ovarian cancer from benign/normal ovaries. Both quantification and spatial distribution of hemoglobin contributed significantly. Invasive epithelial cancers exhibited a significantly higher mean relative HbT (rHbT) and displayed an expansive rHbT distribution. By contrast, benign/normal ovaries exhibited scattered distribution. Moreover, the average ${\mathrm{SO}}_{2}$ in invasive epithelial cancers, borderline tumors, and stromal tumors was significantly reduced compared with that in benign or normal ovaries [Fig. 2(b)].^42^

Further enhancement in classification efficacy was achieved through the application of a generalized linear model and support vector machines, increasing AUC from 0.89 to 0.92, and 0.92 to 0.93, respectively.^51^ Addressing measurement errors, researchers investigated a multipixel method for rHbT and ${\mathrm{SO}}_{2}$ estimation. Machine learning models based on support vector machine models trained with significant features achieved an AUC of 0.93 in distinguishing malignant from benign/normal tumors.^52^ Moreover, a UNet model, enhanced by US, was developed to improve the accuracy of reconstructing optical absorption distributions within lesions, resulting in an AUC of 0.94.^53^

### 2.3 Thyroid Nodule

Epidemiological investigations estimate that 4% to 7% of adults have palpable thyroid nodules, with imaging studies identifying nodules up to 10 times more frequently.^54^ Although thyroid nodules are relatively common, thyroid cancer is rare, representing ∼3% of all malignancies.^25^ Clinically, the primary difficulty lies in distinguishing patients with malignant nodules from those with benign nodules. MSOT exhibits the ability to resolve both intranodular microvasculature and extrathyroidal blood vessels, which was verified by histopathology.^55^ Researchers use multiparametric PA imaging to classify papillary thyroid carcinoma (PTC) nodules.^56^ The PTC nodule had lower ${\mathrm{SO}}_{2}$ compared with benign nodules, aligning with the hypothesis of tumor-related hypoxia [Fig. 2(c)].^43^^,^^56^ A new scoring system in combination with the American Thyroid Association Guideline achieved 83% sensitivity and 93% specificity, potentially reducing unnecessary biopsies in individuals with thyroid nodules.^56^

### 2.4 Skin Cancer

Skin cancer ranked as the fourth most prevalent new cancer globally.^25^ Employing 2D and 3D handheld scanners, MSOT efficiently distinguished tumors and their oxygenation characteristics from normal skin.^44^^,^^57^^,^^58^ Measurements of the tumor dimension through MSOT correlated well with histology findings from excised tumors.^44^^,^^59^ Real-time 3D imaging could offer insights into the morphology of lesions and the associated neo-vasculature, serving as an indicator of tumor aggressiveness^44^ [Fig. 2(d)].

### 2.5 Surgical Guidance and Therapeutic Efficacy Prediction

PA imaging has been investigated as a method of improving surgical guidance, with a specific focus on improving tumor localization and minimizing damage to non-tumor structures such as nerves and vasculature.^60^ This potential application has been explored in a range of tumor types, including pancreatic cancer^61^ and pituitary tumors.^62^

Moreover, researchers exploited PA imaging-derived features, notably tumor oxygenation, for predicting treatment responses in cancer patients. Patient-derived xenograft (PDX) models can be used to assess treatment outcomes.^63^^–^^67^ In the cases of head and neck cancer^64^ and triple-negative breast cancer^66^ PDX models, the identification of a rise in tumor ${\mathrm{SO}}_{2}$ after radiotherapy detected by PA imaging was linked to a notable suppression in tumor growth. The significance of alterations in ${\mathrm{SO}}_{2}$ levels as a predictive factor for treatment response has been highlighted in breast cancer cases treated with Bevacizumab^67^ and in pancreatic cancer cases responding to suboptimal Cabozantinib.^68^ In conclusion, PA imaging may be a straightforward, noninvasive, and cost-effective approach for anticipating therapy efficacy.

## 3 Autoimmune and Inflammatory Diseases

### 3.1 Rheumatoid Arthritis, Psoriatic Arthritis, and Osteoarthritis

**Fig. 3:**
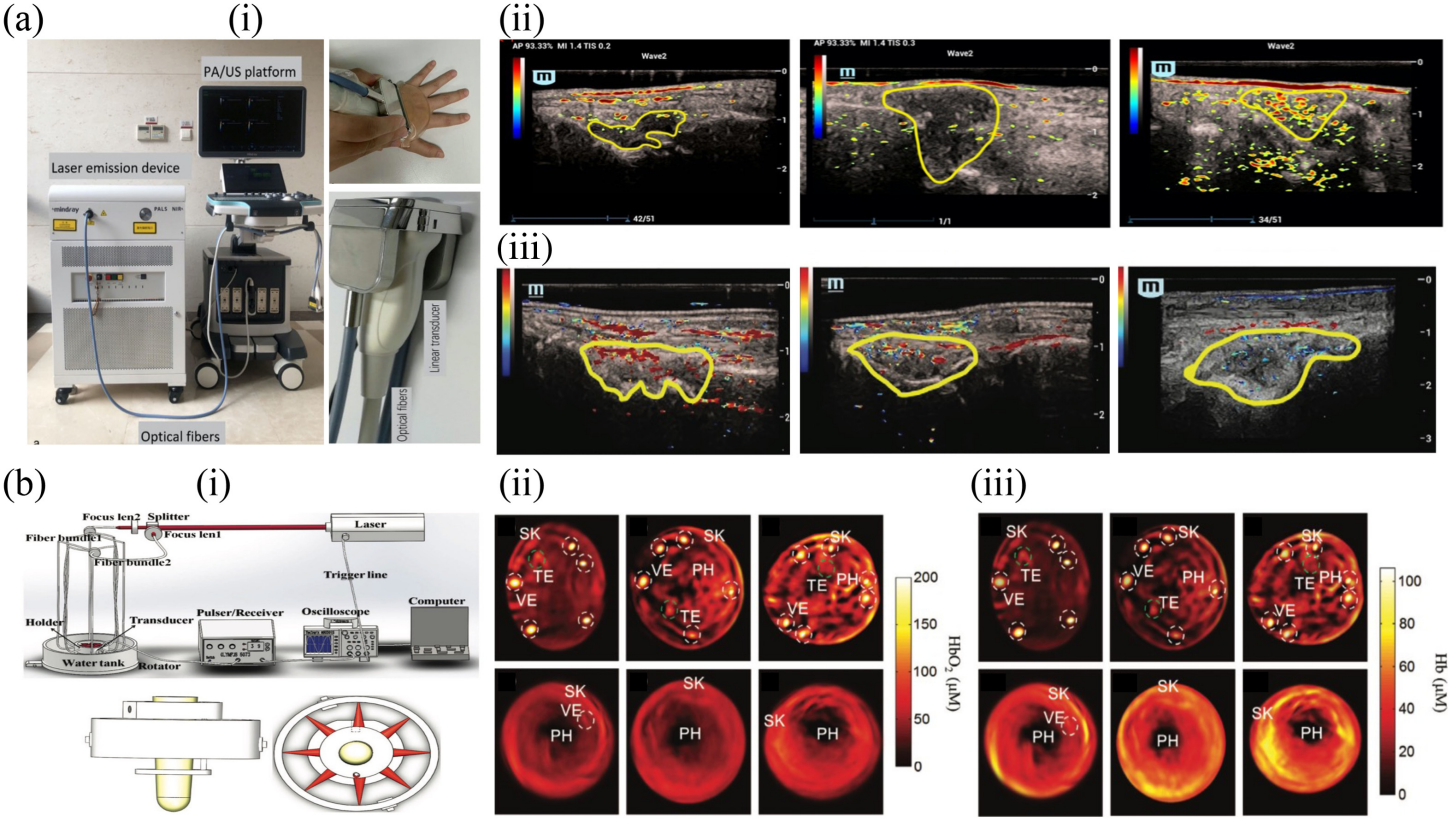
Representative *in vivo* PA imaging for RA and SSc. (a) PA imaging for RA patients: (i) Photograph of the PA/US system, comprising ultrasonic equipment, a handheld linear PA/US probe, and an optical parametric oscillator tunable laser. (ii) PA examples in the semi-quantic system in thickened synovium: PA 1 (no significant signal, left), PA 2 (PA signals in both the center and margin areas), and PA 3 (abundant PA signals). (iii) ${\mathrm{SO}}_{2}$ groupings in thickened synovium, including hyperoxia (left), intermediate status (middle), and hypoxia (right). (b) PA imaging for SSc patients and healthy volunteers: (i) Experimental setup of the PA imaging system and finger holder schematic. (ii) and (iii) The reconstructed ${\mathrm{HbO}}_{2}$ and Hb map for healthy volunteers (top) and SSc patients (bottom). TE, tendon; VE, blood vessels; PH, phalanx; SK, skins. Panels a-i and a-ii are reprinted with permission from Ref. 76, under the terms of Creative Commons CC BY license. Panel a-iii is reprinted with permission from Ref. 77 © RSNA. Panel b is reprinted with permission from Ref. 78 © Wiley.

Rheumatoid arthritis (RA) is a common chronic inflammatory disorder, afflicting around 0.5% to 1% of the general population globally.^69^ It is characterized by progressive inflammation of synovial leading to degradation of cartilage, erosion of bone, and subsequent disability.^70^ US synovitis scoring provides an accessible, secure, and widely employed tool for assessing and monitoring RA’s disease progress.^71^ However, gray-scale US might not comprehensively visualize inflamed synovium, and power Doppler US exhibits limitations in detecting microvessels.^72^^,^^73^ Consequently, researchers are exploring the utility of PA in detecting subtle inflammatory lesions in RA. Metacarpophalangeal joints of RA patients revealed pronounced hyperemia and hypoxia in inflamed synovium, indicative of heightened metabolic demand and relatively inadequate oxygen supply.^74^ Elevated PA signals were detected in RA patients who had evident synovitis, and there was a strong correlation with power Doppler US findings.^75^ Zhao et al.^76^ developed a semi-quantic PA/US scoring system evaluating seven small joints in RA patients. This PA score exhibited strong positive correlations with key clinical disease activity indices of RA [Fig. 3(a)].^76^ Classification of thickened synovium into hyperoxia, intermediate oxygenation status, and hypoxia based on ${\mathrm{SO}}_{2}$ values’ coloration revealed insightful distinctions. RA patients with hyperoxic synovium displayed a greater abundance of vasculature depicted by Doppler US in comparison with those with hypoxic and intermediate synovium. Individuals with an intermediate oxygen status had lower clinical disease activity indices when compared with those with hypoxia. Notably, the identification of hypoxia within thickened synovium through PA imaging was associated with reduced vascularization and increased disease activity in RA [Fig. 3(a)].^77^

Psoriatic arthritis (PsA) is a heterogeneous inflammatory disorder involving joint, entheses, or skin/nail psoriasis.^79^^,^^80^ The first step in PsA patent management is the precise and prompt diagnosis that enables timely therapeutic interventions, primarily focusing on the recognition of inflammatory musculoskeletal disease manifestations.^80^ Capitalizing on its high sensitivity to inflammation, researchers use MSOT to scrutinize finger joints in PsA patients. The semi-quantitative evaluation of the ${\mathrm{HbO}}_{2}$ and Hb PA signals demonstrated that blood content and oxygenation were significantly elevated in PsA patients when compared with individuals from healthy group. This observation underscored the potential of MSOT in the early stage detection of PsA-associated inflammation.^81^

Osteoarthritis (OA) is the most common joint disorder mainly impacting the diarthrodial joints.^82^ Evolving from a cartilage-limited perspective, the pathophysiology conception now embraces OA as a multifactorial disease affecting the entirety of the joint structure.^83^ In a pilot study, researchers employed multispectral quantitative PA tomography to investigate distal interphalangeal joints of OA patients. Compared with normal joints, OA-affected joints displayed significantly elevated water content, decreased ${\mathrm{SO}}_{2}$, and increased acoustic velocity.^84^

### 3.2 Systemic Sclerosis and Raynaud’s Phenomenon

Systemic sclerosis (SSc) is an autoimmune-driven connective tissue disorder identified by microangiopathy and subsequent widespread systemic fibrosis.^85^^,^^86^ In the early stage of the disease, vascular abnormalities become evident in small blood vessels, and eventually advance to obliterative vasculopathy, leading to issues such as tissue hypoxia, oxidative stress, and vascular complications.^86^ Raynaud’s phenomenon (RP), marked by pronounced peripheral vasospasm triggered by cold or emotional stress, can lead to severe outcomes including amputation. RP might be primary or secondary, as observed in SSc and other conditions.^87^ The inconspicuous alteration in the nail fold microscopic patterns in early SSc patients may find resolution through PA, potentially addressing the clinical imperative for early-stage diagnosis.

Early stage SSc and established SSc patients exhibited significantly diminished ${\mathrm{SO}}_{2}$ levels and pronounced skin thickening, distinguishing them from individuals afflicted by primary RP or healthy volunteers [Fig. 3(b)].^78^^,^^88^^,^^89^ Similar results have been obtained using MSOT/US. ${\mathrm{HbO}}_{2}$ and HbT of subcutaneous finger tissue measured by MSOT were significantly lower in SSc patients in comparison with those in healthy volunteers.^90^ Although the vascular volume remained similar between SSc and healthy control patients, oxygenation within the digital arteries was reduced in SSc patients, indicative of functional impairment. The increase in oxygenation after occlusion release was significantly reduced in individuals with SSc in comparison with the healthy control.^89^ Utilizing PA, the differentiation of SSc-RP from healthy volunteers achieved a baseline AUC of 0.72, with elevated diagnosis accuracy observed post-cold stimulus (5 min: AUC=0.89, 15 min: AUC=0.91).^91^ Furthermore, significantly lower MSOT values in progressive SSc patients compared with those with stable disease were observed, indicating the potential of MSOT to evaluate the disease activity of SSc and monitor therapeutic responses.^90^

### 3.3 Giant Cell Arteritis

Giant cell arteritis (GCA) is the most common systemic vasculitis in adults.^92^ Prominent manifestations include headache, jaw claudication, and visual symptoms, with irreversible vision loss being the most severe complication.^93^ Temporal artery biopsy is the gold diagnostic standard for GCA. Noninvasive PA methods are under investigation for early GCA diagnosis. Spectral analysis and the pixel-classification algorithm generated images that closely resembled the findings observed in histopathological examinations. By utilizing minimally supervised spectral analysis, images resembling histology were produced, providing clear delineation between the artery wall and the lumen. Moreover, the PA spectra were successfully distinguished between individuals with biopsy-confirmed GCA and controls.^94^

### 3.4 Inflammatory Bowel Disease

Idiopathic inflammatory bowel disease is a chronic systemic inflammatory disease predominantly affecting the gastrointestinal tract, encompassing Crohn’s disease (CD) and ulcerative colitis (UC).^95^ Uncontrolled inflammation is associated with higher complication rates and mortality, which prompts the exploration of novel noninvasive diagnostic and assessment modalities.^96^ Researchers used MSOT to evaluate intestinal inflammation in CD patients. US-guided MSOT enabled rapid and noninvasive evaluation of HbT, ${\mathrm{HbO}}_{2}$, and Hb content within the intestinal wall. This approach allowed for the quantification of tissue perfusion and oxygenation, which were dependent on hemoglobin and reflected the impact of inflammation.^97^ Significant distinctions in measurements at 760 nm and unmixed HbT were observed between remission CD patients identified by endoscopic and active CD patients.^98^ In pediatric patients with CD and UC, HbT signals exhibited a significant increase in the terminal ileum of CD patients and in the sigmoid colon of UC patients. There parameters correlated well with the disease activity, highlighting the potential for noninvasive evaluation in pediatric patients with IBD.^99^

## 4 Endocrinology and Metabolism Disease

### 4.1 Diabetes Mellitus

Diabetes mellitus (DM) is a prevalent health issue in the 21st century, presenting a significant public health challenge.^100^ DM-related complications include both macrovascular and microvascular conditions, including coronary heart disease, peripheral arterial disease, diabetic kidney disease, and retinopathy.^101^

**Fig. 4:**
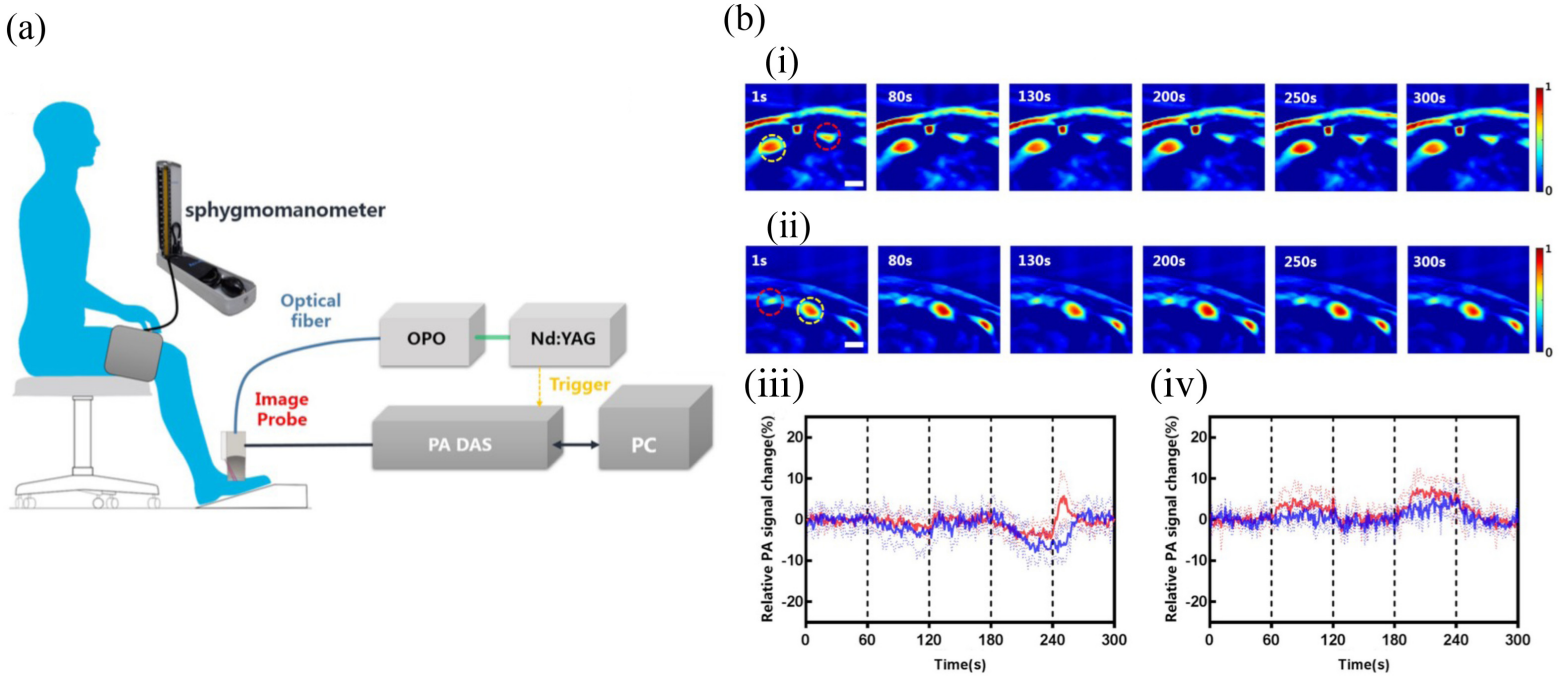
Representative *in vivo* PA imaging for DM. (a) Schematic of PA tomography system. A sphygmomanometer was used to induce occlusion. PC, personal computer; ND:YAG, laser; OPO, optical parametric oscillator; DAS, data acquisition system. (b) PA imaging during occlusion in healthy and DM patients. (i) and (ii) Sequences of PA images following occlusion in healthy volunteers and DM patients, respectively. (iii) Hemodynamic changes in arteries. (iv) Hemodynamic changes in veins. Blue line: DM; red line: healthy volunteer. Figure 4 is reprinted with permission from Ref. 106 © Wiley.

PA imaging, capable of monitoring perfusion changes,^102^ and measuring ${\mathrm{SO}}_{2}$ in artery or capillary vessels,^19^^,^^103^^–^^105^ hold promise for staging DM severity via quantitative imaging of vascular complications. Researchers found that, after occlusion, DM patients exhibited a distinctive peripheral hemodynamic response and had lower ${\mathrm{SO}}_{2}$ levels than healthy individuals, highlighting the capacity of PA imaging to detect vascular dysfunction of DM (Fig. 4).^106^ In addition, MSOT emerged as a tool to visualize peripheral vasculature and assess flow-mediated dilation, which can characterize macrovascular endothelial function—a biomarker for early stage atherosclerosis accentuated in DM.^107^ MSOT also displayed potential in imaging peripheral arterial disease, revealing significantly reduced ${\mathrm{HbO}}_{2}$, HbT, and ${\mathrm{SO}}_{2}$ compared with healthy volunteers.^108^ Furthermore, MSOT facilitates the assessment of endocrine and metabolic functions by visualizing lipids and adipose tissue. Reber et al.^109^ observed differences in brown adipose tissue composition between DM and healthy volunteers.

Among serious complications associated with DM, poor wound healing, persistent ulceration, and the subsequent need for limb amputation pose significant challenges.^110^ Previous studies demonstrated the utility of PA imaging in monitoring wound healing. A case study showed that higher perfusion and ${\mathrm{SO}}_{2}$ were correlated with surgical observations. In a cohort, radial artery oxygenation increased significantly after hyperbaric oxygen therapy.^111^ Similar outcomes emerged from studies measuring ${\mathrm{SO}}_{2}$ following topical hemoglobin treatment, with increased ${\mathrm{SO}}_{2}$ predicting treatment response.^112^ Moreover, PA imaging can directly visualize angiogenesis.^113^ Therapy-responsive patients exhibited evident indications of angiogenesis and a significantly increased rate of PA signals. Negative correlations were detected between the intensity of PA signals and the size of the wound. An increased rate of PA signal or angiogenesis could predict the time of healing within the first 30 days of monitoring.^113^

### 4.2 Thyroid Disease

The thyroid gland is an important endocrine organ, producing thyroxine to regulate the organ function, metabolic condition, and protein synthesis rate. For imaging of thyroid functional pathology such as Graves’ disease (hyperthyroidism) or Hashimoto thyroiditis (hypothyroidism), existing modalities involve assessing the blood flow via Doppler US or tracking the uptake of radioactive contrast agents.^114^ PA could reveal microvessels in the human thyroid that escape Doppler US.^115^ Further, MSOT could image lipids and water, alongside vascularization and oxygenation predicted on hemoglobin.^19^ In Graves’ disease patients, ${\mathrm{HbO}}_{2}$ and HbT were significantly elevated while fat content exhibited significant reduction, distinguishing them from healthy controls.^43^ These initial investigations underscore the capability of PA imaging to offer rapid and radiation-free functional thyroid imaging.^19^

## 5 Future and Challenges

Over the past decades, PA imaging has demonstrated significant potential in clinical applications, as outlined in this review. The next decade is poised to witness a substantial increase in the utilization of PA imaging in clinical practice, owing to its advantages in penetration depth, functional imaging capabilities, multimodality integration, and safety. However, several challenges remain for the incorporation of PA imaging into routine medical practice.

First, the primary challenge lies in the limited penetration depth.^116^ Although PA imaging provides enhanced depth compared with purely optical imaging techniques, inherent attenuation of light energy restricts its penetration to ∼3  cm *in vivo* clinical settings.^117^^,^^118^ Consequently, imaging internal organs such as the heart, liver, pancreas, and uterus in humans remains a challenging task. The exploration of advanced laser technologies and advanced signal processing techniques may help increase the penetration depth. Second, the differentiation of imaging contrast is important. Although endogenous contrasts are various, such as hemoglobin, water, lipid, and melanin, their absorption spectra often overlap, posing difficulties for algorithms to distinguish them, even with MSOT [Fig. 1(b)].^119^ This limitation hinders the accuracy of signal quantification and brings uncertainty to PA imaging.^120^^,^^121^ Investigating contrast agents and employing deep learning algorithms may fascilitate the differentiation of imaging contrasts.^122^^,^^123^ Third, the absence of standardized protocols is another critical concern. Unlike mature imaging modalities such as MRI and CT, PA imaging lacks standardized procedures and quality control measures.^121^^,^^124^ This can introduce variability in image quality and interpretation, making it a great challenge to compare results across various studies, institutions, and devices.^7^ Establishing education and training procedures for data acquisition and interpretation is imperative. Finally, the deficiency in large-scale clinical validation poses an obstacle. The reliability, accuracy, and value of PA imaging for specific diagnosis and evaluation need to be thoroughly established through well-designed clinical trials.^124^

## 6 Conclusions

Despite extant challenges, PA imaging exhibits significant promise for both research and clinical applications across diverse fields such as tumors, autoimmune diseases, inflammatory conditions, endocrine disorders, and so on. From bench to bedside, PA imaging aims to facilitate the diagnosis, evaluation, treatment, and prognosis anticipation. Through sustained collaborative efforts among researchers, clinicians, and technologists, PA imaging may pave the way for personalized patient management. As a result, it has the potential to transition from an uncommon technology to a routine strategy, contributing to more precise medical approaches in the foreseeable future.

## Data Availability

Data sharing is not applicable to this review, as no new data were created or analyzed.
